# Supplementary material for: Upstream Stimulatory Factors Regulate HIV-1 Latency and Are Required for Robust T Cell Activation
Source: Viruses. 2023 Jun 28;15(7):1470. doi: 10.3390/v15071470 (PMC10384547; doi:10.3390/v15071470)

Legends to Supplementary Figures

Figure S1. shRNA knockdown of *USF2* inhibits HIV-1 expression. A, C: Jurkat mHIV-Luciferase cells were transduced with an pLKO empty vector control or shRNA targeting USF1 (A) or USF2 (C). Transduced cells were selected for with 3 μg/mL puromycin for up to 8 days after which lysates were analyzed by immunoblotting using antibodies against USF1, USF2, and Tubulin. **B, D:** USF1 (B) or USF2 (D) shRNA transduced mHIV-Luciferase cells were left untreated (Ve, DMSO) or stimulated with 5 nM PMA for 4 hours prior to measuring luciferase activity (n = 3, mean ± SD).

Figure S2. ***USF2* knockout limits proviral induction in TZM-bl cells. A:** *USF2* KO in the TZM-bl cell line was produced by using CRISPR-Cas9. Immunoblotting of cellular lysates was performed using antibodies against USF2 and Tubulin. **B:** Wildtype or *USF2* KO TZM-bl cells were left untreated (Ve, DMSO) or incubated with 5 nM PMA for 4 hours prior to measuring luciferase activity. The relative light units (RLU) are displayed (n = 3, mean ± SD).

Figure S3. Reincorporation of USF1 or USF2 rescues LTR transcription in *USF2* KO cells. A: Schematic representation of the LAI LTR-Tat-IRES-GFP reporter construct as well as the CMV-EV-EF1α-RFP (EV), CMV-USF1-EF1α-RFP (USF1), and CMV-USF2-EF1α-RFP (USF2) expression vectors. **B:** Depiction of the flow cytometry scatter plot generated following co-transfection of LTR reporter and RFP expression vector. The bottom left quadrant contains un-transfected cells, negative fluorescent cells. The top left population is co-transfected by the

LTR reporter and the RFP expression vector but the LTR is silent. Cells in the top right quadrant are co-transfected by transcriptionally active LTR reporter as well as the RFP expression vector. The bottom right quadrant are cells transfected only by the active LTR reporter. **C:** Representative scatter plots following co-transfection of wildtype or *USF2* KO HEK293T cells with the LTR-Tat-IRES-GFP and either CMV-EV-EF1α-RFP, CMV-USF1-EF1α-RFP (USF1), or CMV-USF2-EF1α-RFP (USF2) vector.

Figure S4. Loss of USF1 or USF2 does not affect cellular growth. A, B: Equivalent number of Wildtype, *USF1* KO (A), and *USF2* KO (B) Jurkat mHIV-Luc cells were plated, and cell number counted on the indicated day (n = 3, mean ± SD). **C, D:** As in A, B but cellular viability was determined (n = 3, mean ± SD).

Figure S5. USF2 regulates HIV expression regardless of Tat. A: Schematic representation of the CMV-EV-EF1α-RFP vector and the LAI LTR-IRES-GFP (-Tat) and LTR-Tat-IRES-GFP (+Tat) reporter constructs. **B:** Depiction of the flow cytometry scatter plot generated following co-transfection of LTR reporter and RFP expression vector. See Figure S3*B* for further description. **C, D:** Representative scatter plots following co-transfection of wildtype or *USF2* KO HEK293T cell lines with RFP expression vector and either LTR-IRES-GFP (C) or LTR-Tat-IRES-GFP (D) LAI derived reporter construct.

Figure S6. Loss of either *USF1* or *USF2* impairs expression of genes downstream of multiple T cell activation pathways. A - D: Hallmark GSEA plots of RNA-seq data analyzed

by DESeq2 of PMA/ionomycin stimulated *USF1* KO and *USF2* KO Jurkat T cells. Analysis was performed on n = 3 RNA-seq samples.

Figure S7. USF2 enhances HIV-1 infection of T cells. A: Representative scatter plots of Wildtype, *USF1* KO, *USF2* KO Jurkat Tat T cells 3 days post-infection with Red-Blue-HIV-1 dual reporter virus [57]. LTR driven BFP expression is depicted on the x-axis while mCherry expression driven by the constitutive CMV promoter on the y-axis. **B:** Wildtype, *USF1* KO, or *USF2* KO Jurkat Tat cells were infected at the same multiplicity of infection (M.O.I. ~0.1). 3 days post-infection, cells were analysed by flow cytometry and the infection rate was normalized to the number of wildtype cells that were infected (n = 3, mean ± SD). **C:** Same as in B, but the ratio of productive to latent infection was determined (n = 3, mean ± SD).

Figure S8. Representative flow cytometry gating strategy. A, B: Representative scatter plots depicting the gating strategy employed for HEK293T cell lines (A) and Jurkat T cell lines (B). Homogeneous populations of live cells were isolated and assessed by setting threshold forward scatter (FSC) and side scatter (SSC) settings.

Supplementary Figure 1

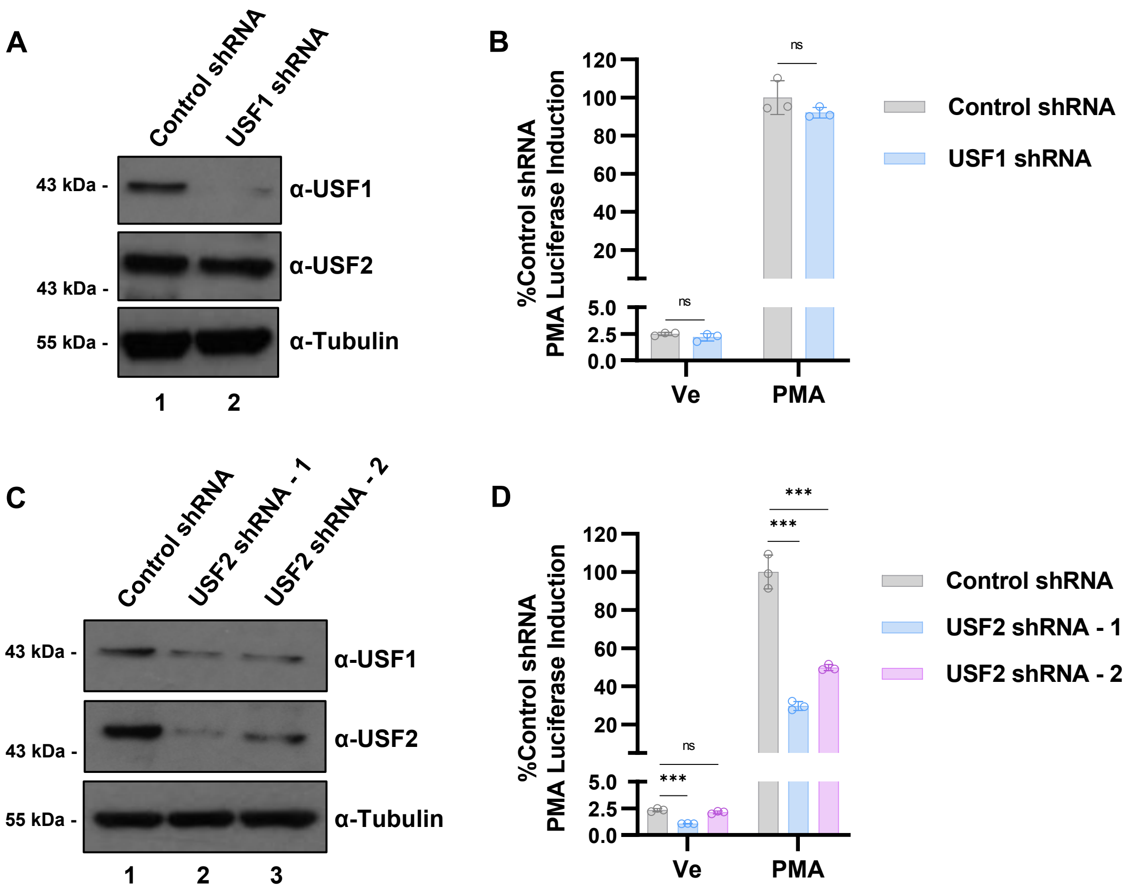

Supplementary Figure 2

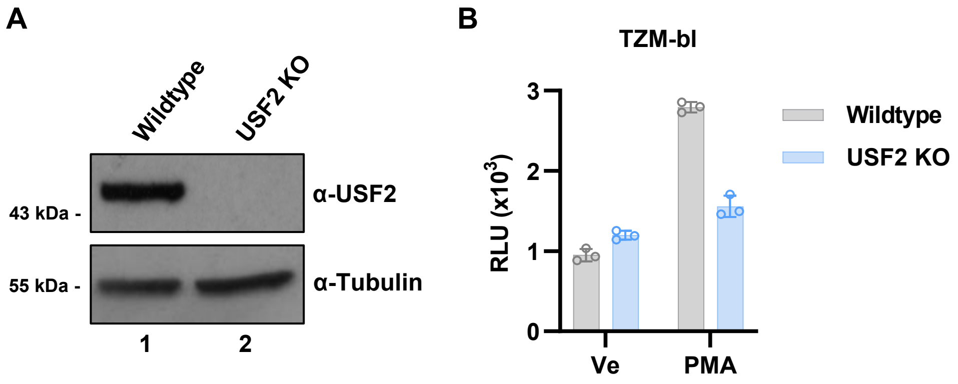

Supplementary Figure 3

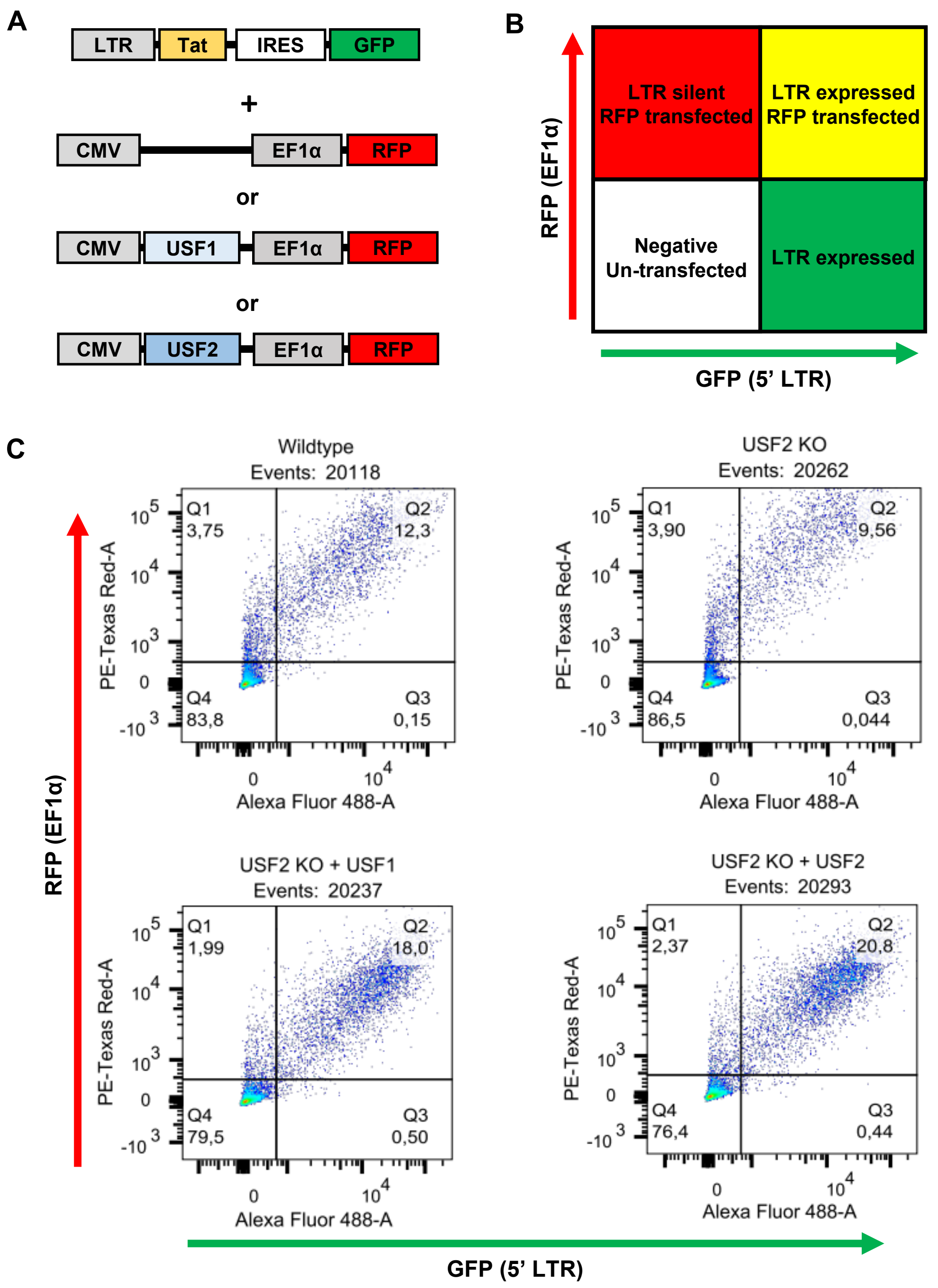

Supplementary Figure 4

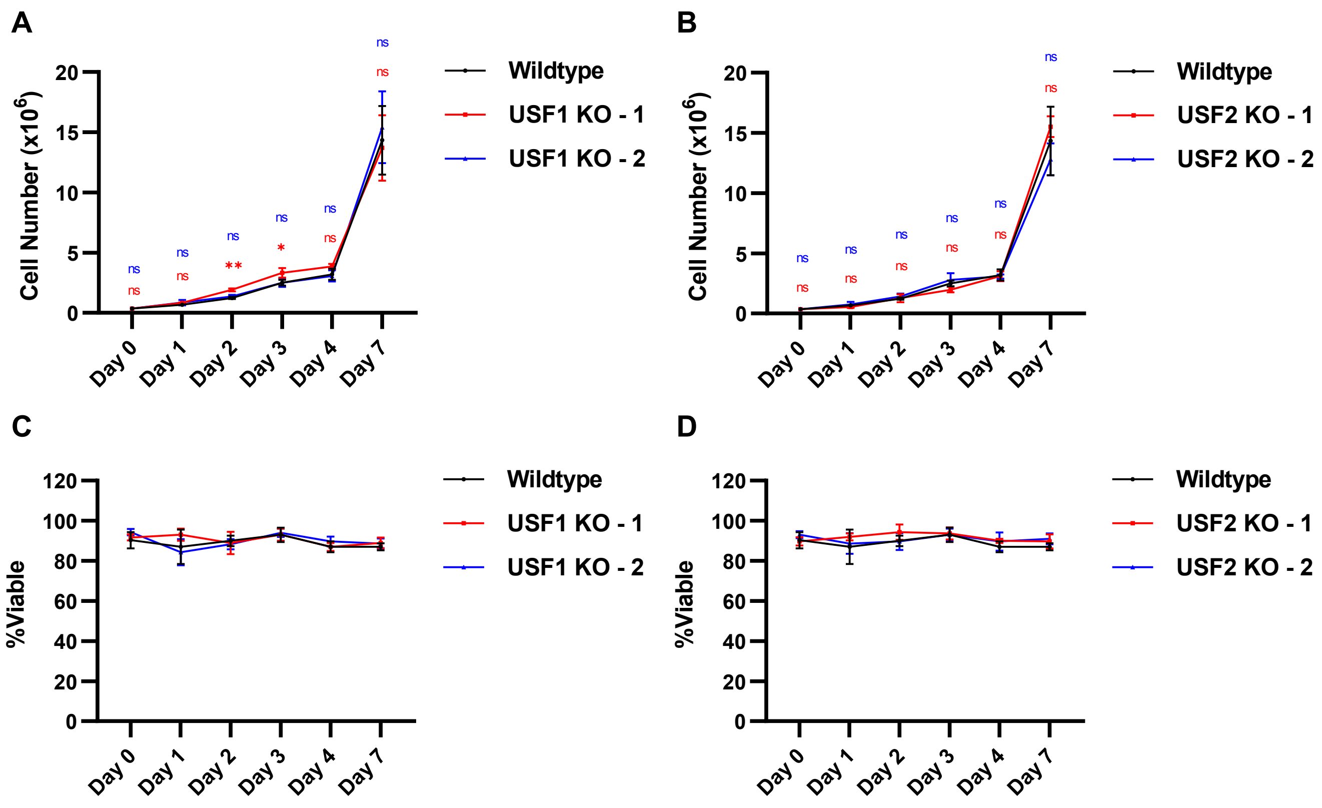

Supplementary Figure 5

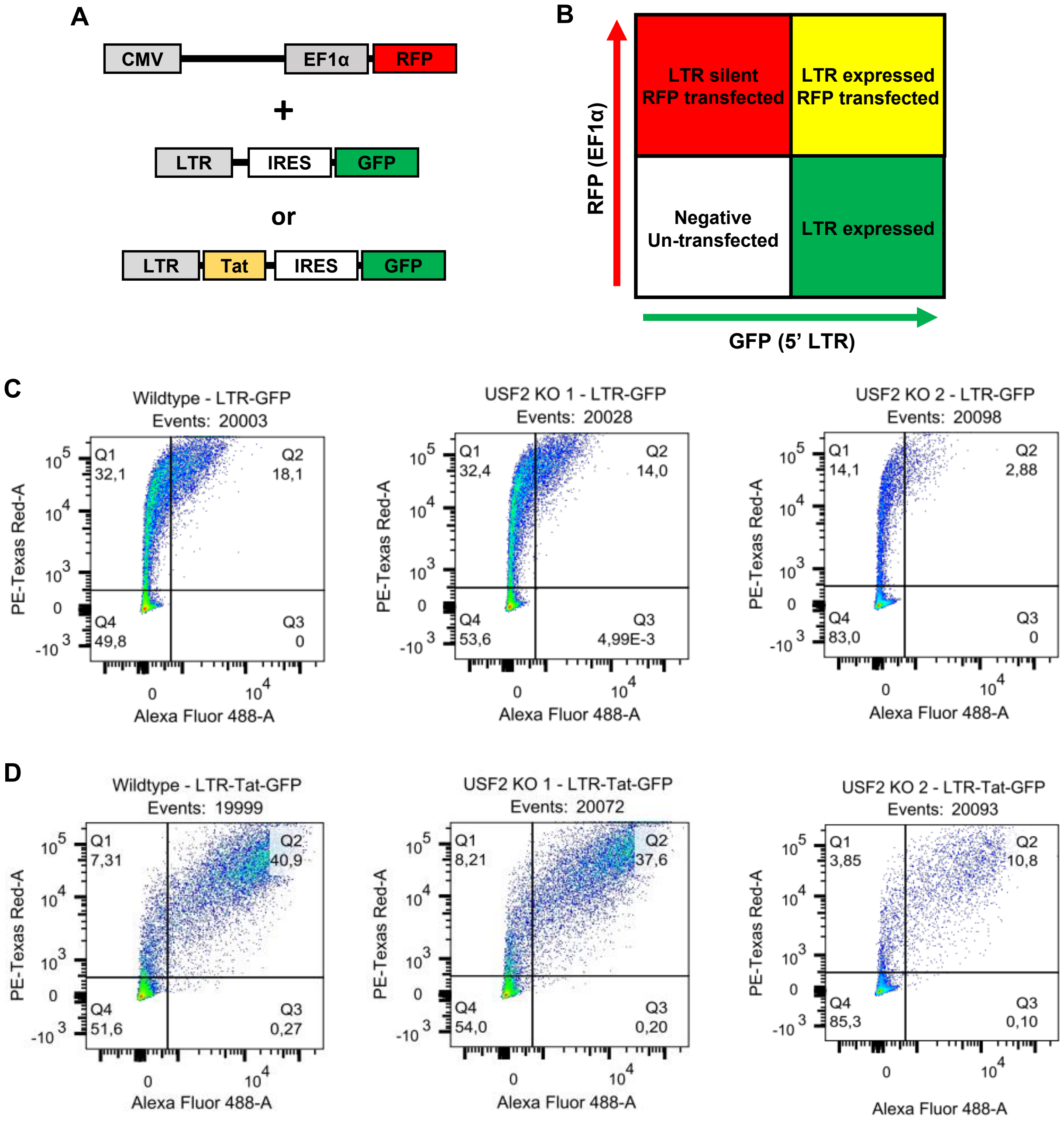

Supplementary Figure 6

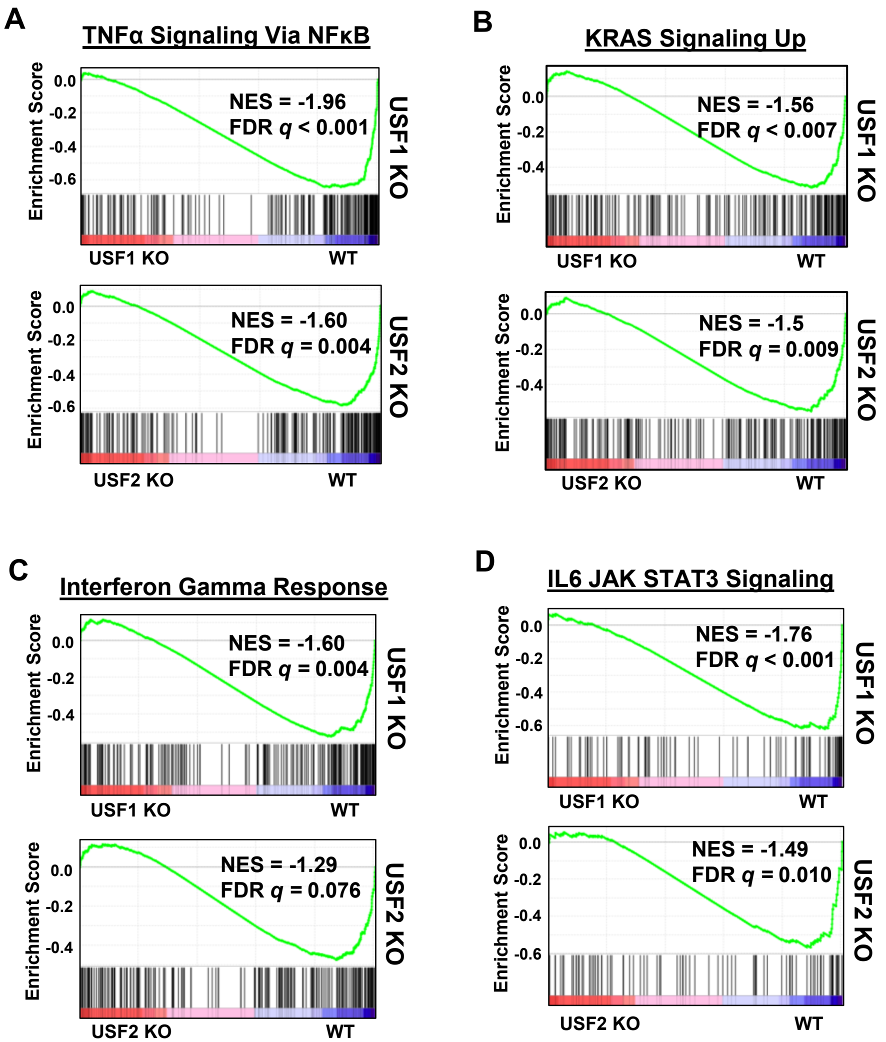

Supplementary Figure 7

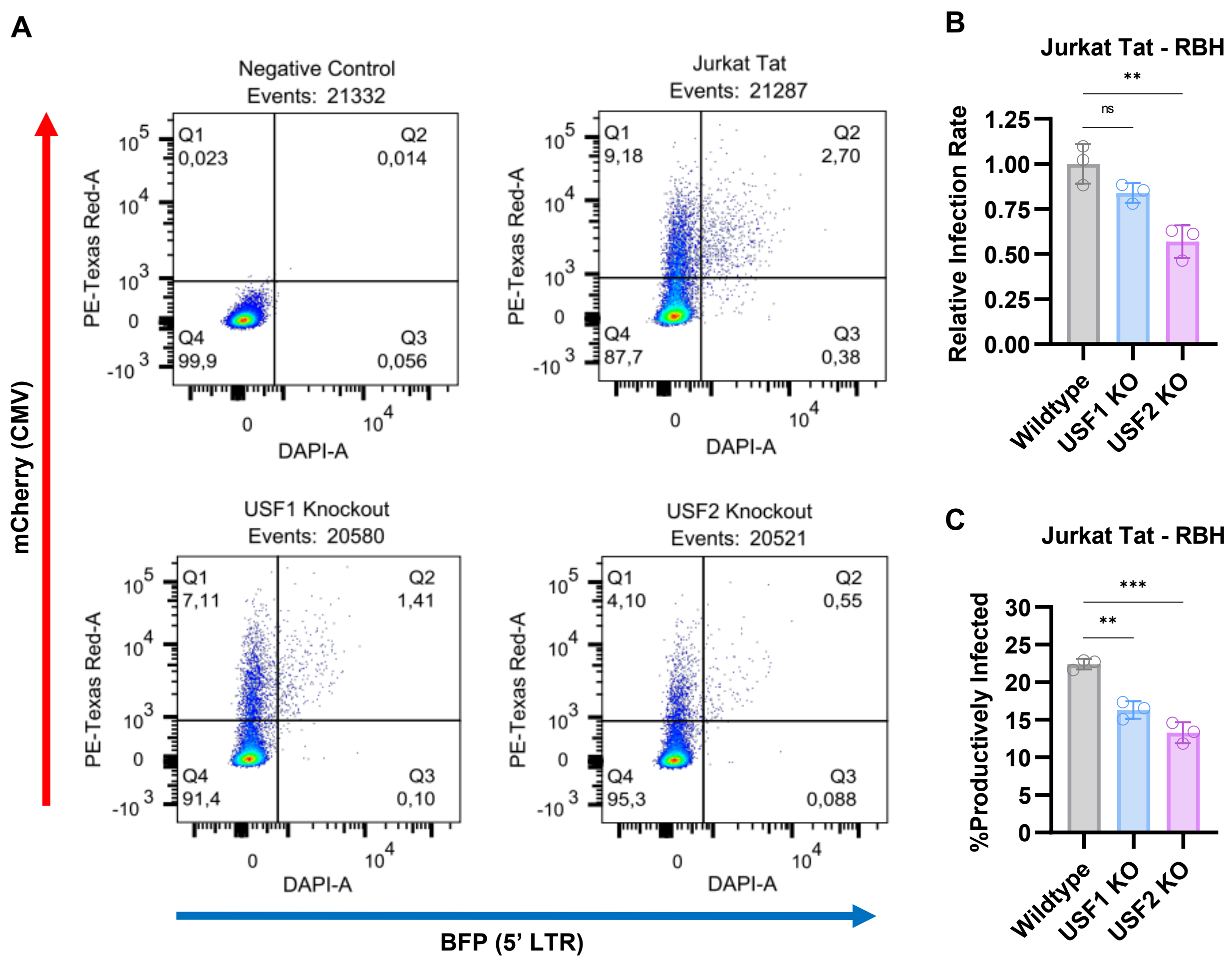

Supplementary Figure 8

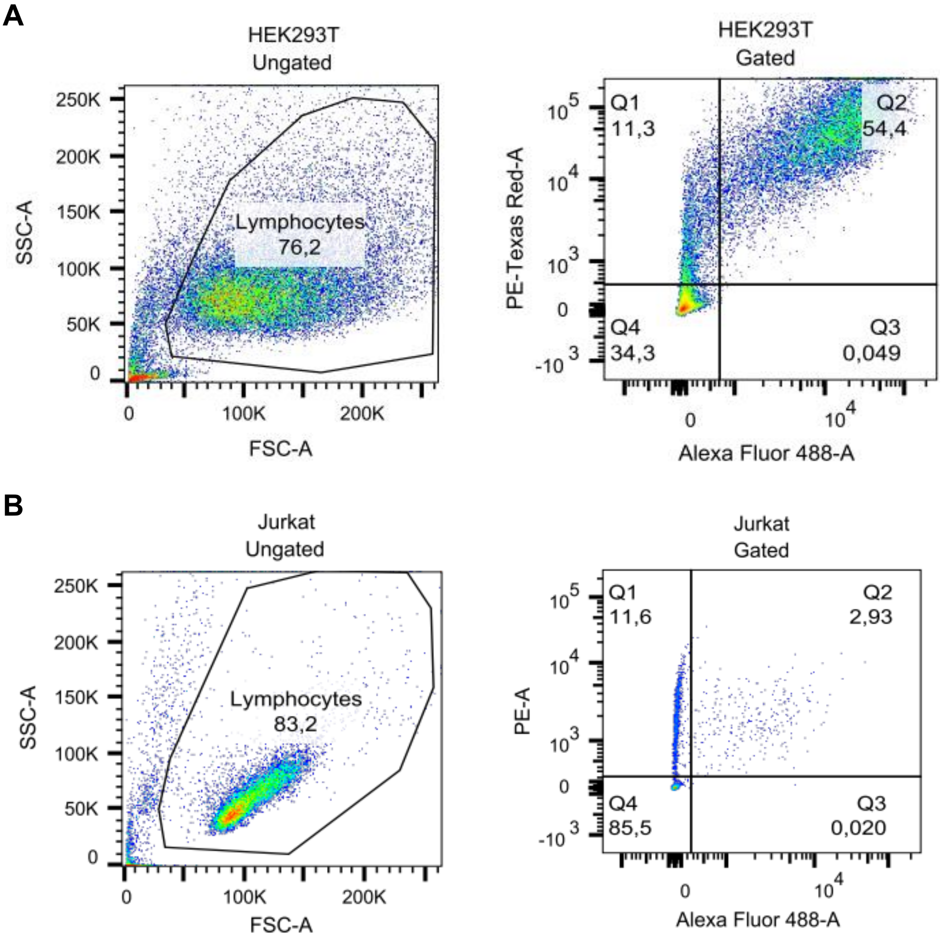

Supplement: Supplementary file 1 [file viruses-15-01470-s001.zip › viruses-2394984-supplementary.pdf]
